# A Mobile Technology for Collecting Patient-Reported Physical Activity and Distress Outcomes: Cross-Sectional Cohort Study

**DOI:** 10.2196/17320

**Published:** 2020-05-04

**Authors:** Miyeon Jung, SaeByul Lee, Jisun Kim, HeeJeong Kim, BeomSeok Ko, Byung Ho Son, Sei-Hyun Ahn, Yu Rang Park, Daegon Cho, Haekwon Chung, Hye Jin Park, Minsun Lee, Jong Won Lee, Seockhoon Chung, Il Yong Chung

**Affiliations:** 1 Korea Advanced Institute of Science and Technology Seoul Republic of Korea; 2 Division of Breast Surgery Department of Surgery University of Ulsan College of Medicine, Asan Medical Center Seoul Republic of Korea; 3 Department of Biomedical Systems Informatics Yonsei University College of Medicine Seoul Republic of Korea; 4 Swallaby Seoul Republic of Korea; 5 Department of Psychiatry University of Ulsan College of Medicine, Asan Medical Center Seoul Republic of Korea

**Keywords:** telemedicine, breast neoplasms, mobile apps, quality of life, validation, patient-reported outcome measures (PROMs), questionnaire

## Abstract

**Background:**

Electronic patient-reported outcome (PROs) provides a fast and reliable assessment of a patient’s health-related quality of life. Nevertheless, using PRO in the traditional paper format is not practical for clinical practice due to the limitations associated with data analysis and management. A questionnaire app was developed to address the need for a practical way to group and use distress and physical activity assessment tools.

**Objective:**

The purpose of this study was to assess the level of agreement between electronic (mobile) and paper-and-pencil questionnaire responses.

**Methods:**

We validated the app version of the distress thermometer (DT), International Physical Activity Questionnaire (IPAQ), and Patient Health Questionnaire–9 (PHQ-9). A total of 102 participants answered the paper and app versions of the DT and IPAQ, and 96 people completed the PHQ-9. The study outcomes were the correlation of the data between the paper-and-pencil and app versions.

**Results:**

A total of 106 consecutive breast cancer patients were enrolled and analyzed for validation of paper and electronic (app) versions. The Spearman correlation values of paper and app surveys for patients who responded to the DT questionnaire within 7 days, within 3 days, and on the same day were .415 (*P*<.001), .437 (*P*<.001), and .603 (*P*<.001), respectively. Similarly, the paper and app survey correlation values of the IPAQ total physical activity metabolic equivalent of task (MET; Q2-6) were .291 (*P*=.003), .324 (*P*=.005), and .427 (*P*=.01), respectively. The correlation of the sum of the Patient Health Questionnaire–9 (Q1-9) according to the time interval between the paper-based questionnaire and the app-based questionnaire was .469 for 14 days (*P*<.001), .574 for 7 days (*P*<.001), .593 for 3 days (*P*<.001), and .512 for the same day (*P*=.03). These were all statistically significant. Similarly, the correlation of the PHQ (Q10) value according to the time interval between the paper-based questionnaire and the app-based questionnaire was .283 for 14 days (*P*=.005), .409 for 7 days (*P*=.001), .415 for 3 days (*P*=.009), and .736 for the same day (*P*=.001). These were all statistically significant. In the overall trend, the shorter the interval between the paper-and-pencil questionnaire and the app-based questionnaire, the higher the correlation value.

**Conclusions:**

The app version of the distress and physical activity questionnaires has shown validity and a high level of association with the paper-based DT, IPAQ (Q2-6), and PHQ-9. The app-based questionnaires were not inferior to their respective paper versions and confirm the feasibility for their use in clinical practice. The high correlation between paper and mobile app data allows the use of new mobile apps to benefit the overall health care system.

**Trial Registration:**

ClinicalTrials.gov NCT03072966; https://clinicaltrials.gov/ct2/show/NCT03072966

## Introduction

The National Comprehensive Cancer Network recommends that whenever a cancer patient visits a doctor, the doctor should screen for distress, which can be managed by clinical practice guidelines. Similarly, the American Society of Clinical Oncology guidelines suggest all that cancer patients should be screened for depressive symptoms at appropriate intervals at the beginning of and after the visit [1]. Several papers reported that the prevalence of depression and anxiety among cancer survivors was 11.6% and 17.9%, respectively [2].

Patient-reported outcome (PRO) measures, defined by the US Food and Drug Administration as “reporting on the health of patients directly from patients,” is becoming common in the medical field [3]. However, conventional distress screening tools are paper-and-pencil questionnaires, which can cause recall bias and do not reflect real-time episodes of distress. In addition, the use of PRO in traditional paper format is not practical for clinical practice due to limitations associated with data analysis and management [4,5]. Therefore, entering PRO data by electronic means (ePRO) was developed as an alternative [6]. Initially, ePRO was developed based on a web platform, thus offering the portability and viability of tools used for health care assessment via mobile phones [7,8].

Current long-term health care monitoring of patients requires the most promising remote monitoring techniques to provide cost-effective quality control [9]. Therefore, models for remote monitoring of patients combined with the selection of ePRO are recommended. This enables self-management of patient care at all treatment stages while improving the quality of life of the patient [10]. The benefit of the clinical use of the mobile phone app is the possibility to accumulate high-quality and reliable data and archive backups to prevent data loss [11].

Although some discrepancies were reported between the paper-and-pencil and electronic versions of the same questionnaires, there is evidence that electronic and paper PROs reflect equivalent outcomes, whereas some reports suggest that the electronic PRO is more accurate [8,12]. Despite the active use of mobile health (mHealth) apps for measuring distress and physical activity, no validated health care apps have been developed. The questionnaire app was developed to address the need for a practical way to group and use distress and physical activity assessment tools. According to the study guidelines proposed by the International Society for Pharmacoeconomics and Outcomes Research, the data obtained from ePRO questionnaires should be comparable or superior to the data from paper-based questionnaires [13].

Therefore, this study aims to validate the app using correlation analysis between the paper-based gold standard and mobile-based new formats using distress and physical activity questionnaires. Our study examined the association between responses collected through a mobile app and the paper-based face-to-face survey. Even though the same questionnaires were used, responses collected through the new format of mobile technology can be different than responses on paper-based questionnaires. This may be because of screen size or not having the constraint of a face-to-face survey.

As a result, if the mobile app survey was completed on the same day, even though the collection method was different, the responses were almost the same. But responses weren’t similar for questions like asking about time spent sitting on the International Physical Activity Questionnaire (IPAQ). In this aspect, we could conclude that estimates of time spent sitting are not easy to answer correctly and not easy to remember, so it varies depending on the format of collection and time zone, even in the same day. Therefore, IPAQ (Q7; sitting time) is difficult to replace through mobile app collection systems.

Beyond validating the mobile responses that were answered on the same day, we also compared them with the average of mobile responses in the days before and after a few days. The reason we should compare them is that the distress thermometer (DT), IPAQ, and Patient Health Questionnaire–9 (PHQ-9) are asking the status of patients for last 7 days, not on the one single day when the survey was placed.

Therefore, we analyzed how the average values of survey inputs from the mobile-based PRO collection system, answered on the 3 or 7 days before and after the paper-based face-to-face survey, were related to the input value from the paper survey. When we want to replace a face-to-face questionnaire with a mobile app in the real world, it may be more effective to use the average value at specific intervals due to the nature of PROs asking about the status of the recent week, not one day. In our analysis, the correlation became weaker as the intervals became longer, but it still showed a significant correlation.

## Methods

### Study Design and Subjects

This cross-sectional study recruited patients who underwent surgery for breast cancer at the Asan Medical Center. Patients were eligible for study participation if they were women between the ages of 20 and 65 years and had Android smartphones compatible with the WalkON app [14], a free activity tracking app modified for this study. Patients who had distant metastasis, recurrent breast cancer, severe medical conditions such as cardiovascular disease, did not know how to use a smartphone, used iOS smartphones, or were on chemotherapy were excluded.

Written informed consent was obtained from all patient subjects at enrollment. The study protocol was approved by the institutional review board of Asan Medical Center (2016-0819). This study was registered at ClinicalTrials.gov [NCT03072966].

### Ethical Approval

All procedures performed in studies involving human participants were in accordance with the ethical standards of the institutional and/or national research committee and the 1964 Helsinki declaration and its later amendments or comparable ethical standards.

### Recruitment and Follow-Up

During the hospital stay after breast cancer surgery, subjects were contacted by a clinical research assistant. After consenting to participate, participants completed the DT, IPAQ, and PHQ-9 paper-based questionnaires (baseline). The assistant helped patients download the Android-based app (WalkON) to the participants’ smartphones. The main purpose of this study was to calculate the Spearman correlation of baseline values of DT, IPAQ, and PHQ-9 between paper-based questionnaire and app-based questionnaire.

At the 3- and 6-month follow-ups, participants were asked to complete the same version of the paper surveys voluntarily. Since there were few voluntary answers, we only used the first survey to validate the correlation between app and paper surveys.

### Smartphone App and App-Based Questionnaire

Swallaby Inc is a mobile health care app company that has developed a health-related smartphone app (WalkON). This app provides users with a platform for tracking their daily steps and creates mobile communities where users can communicate with each other and view each other’s daily step count to get motivated and promote health-related activities.

App-based self-reporting questionnaires were programmed into this app, wherein study participants could answer the app-based questionnaires. We used this app to conduct weekly and biweekly questionnaires for a future study on the development of a distress screening tool. Daily questionnaires were developed and previously reported by the authors and consisted of self-reporting modules for daily anxiety, sleep, and emotion statuses [9,12]. Responses were collected every week for DT and IPAQ and biweekly for PHQ-9 through the app, and push notifications were sent every week from Sunday to Tuesday to subjects’ smartphones. The responses to these three questionnaires (DT, IPAQ, and PHQ-9) were analyzed in this validation study.

### Statistical Analysis

The walking app-based physical activity and stress collecting systems were validated by calculating the Spearman and concordance correlation between the responses to app-based and paper-based questionnaires. The baseline paper survey was administered once at the recruitment stage. Thereafter, participants voluntarily reported their physical activity and stress through the app. For DT and IPAQ, the survey required participants to record their average stress level and physical activity level scores, respectively, during the last 7 days. For PHQ-9, the survey required participants to record their subjective perception of average life quality level during the last 14 days.

We expected the values collected via paper and mobile app to be almost the same if they were reported on the same day. To infer that the new channel for patient-reported outcome collection, a mobile app, works well, the correlation between two values reported on the same day should be high.

However, since the patients responded to DT and IPAQ at least once per week and PHQ-9 biweekly on any day, the subsample of patients who responded to the questionnaires on the same day (recruitment date) was small.

Thus, to investigate the validity of the mobile app survey, we also calculated the correlation between two values: the values at the baseline date and values reported on a few days around the baseline date. More specifically, we picked the values recorded on the baseline date ±3 days and calculated the correlation between the values reported on and those reported around the baseline date. Similarly, we calculated the correlation between the values recorded within and after 7 days from the baseline date. For the PHQ-9 only, we also calculated the correlation between the values recorded within and after 14 days from the baseline date. Here, few participants (less than 10%) reported each value more than two times within the weekly and biweekly periods. In such cases, we calculated the average value of the reported numbers within the defined period.

Taken together, for DT and IPAQ, we first calculated the average of the values reported on the same day, within 3 days, and within 7 days. Then, we calculated the Spearman correlation between the average value in the mobile app and the paper survey answer. For PHQ-9, we calculated the average of the values reported on the same day, within 3 days, within 7 days, and within 14 days.

In addition to the Spearman correlation, we also calculated the required sample size and actual statistical power using G*Power software, given the value of α as .05, the power (1-β) as .95, and the Spearman correlation value.

We also conducted a robustness check on our correlation analysis by calculating the bias correction factor of the concordance correlation coefficient (CCC) [15] and by positing an ordered logistic regression model. For the CCC, previous studies suggest the following descriptive scale for values (for continuous variables): <.90 poor, .90 to .95 moderate, .95 to .99 substantial, and >.99 almost perfect correlation [15]. For the ordered logistic regression model, dependent variables were the discrete variables of DT, IPAQ and PHQ in the paper survey, and independent variables were the continuous variables of average DT, IPAQ, and PHQ in the mobile app survey. We calculated the coefficients on the values of mobile app survey and their odds ratios.

## Results

### Patient Characteristics

**Figure 1:**
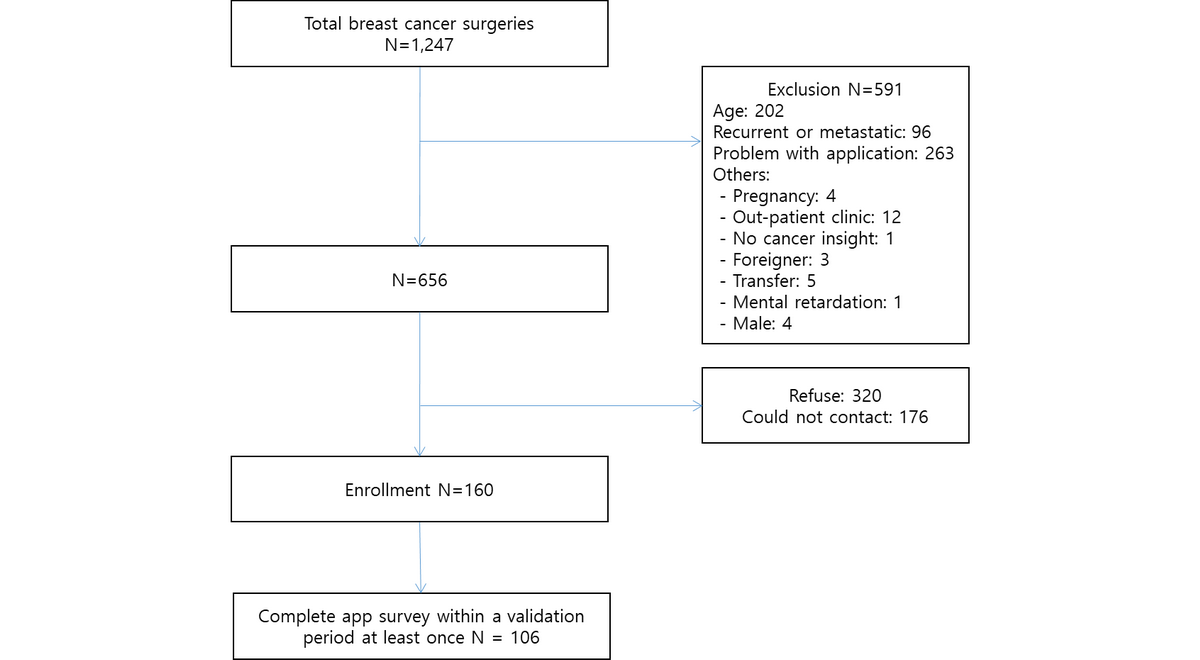
Participant enrollment.

**Figure 2:**
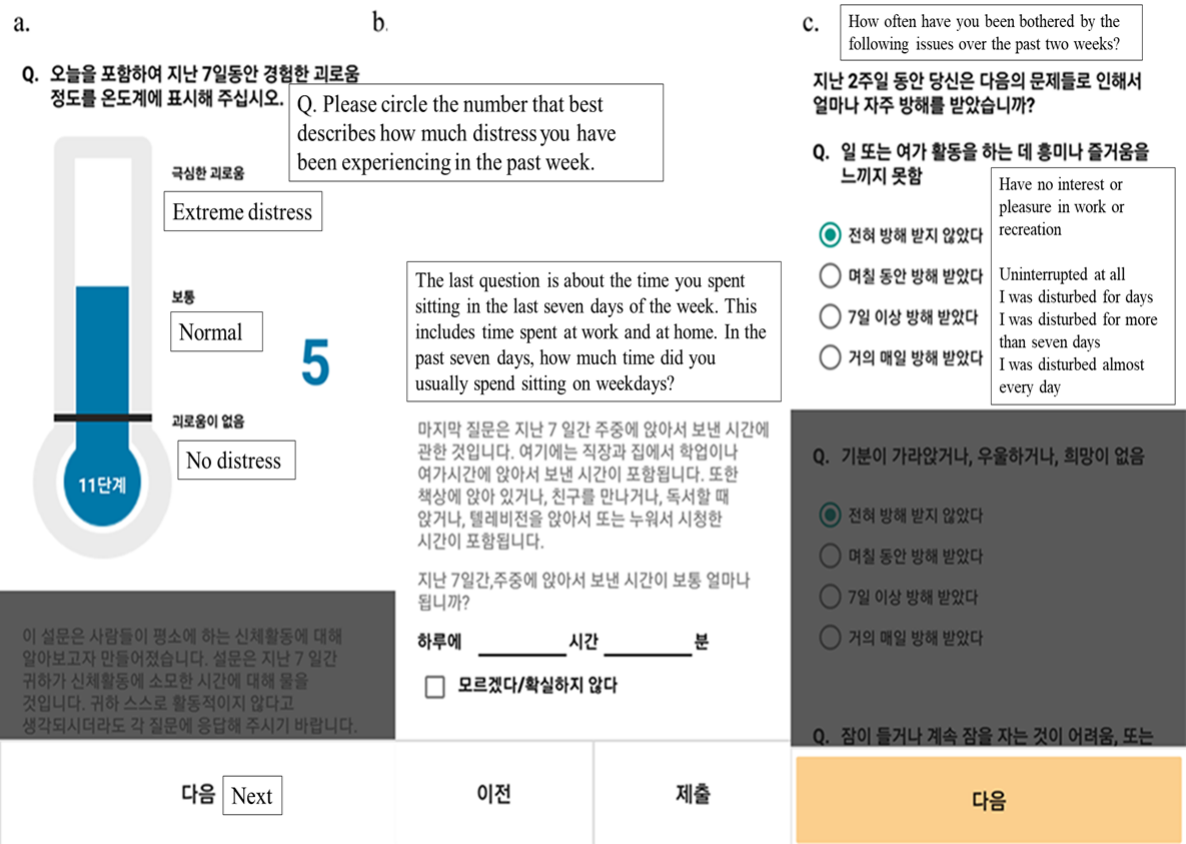
App screenshots of the (A) Distress Thermometer, (B) International Physical Activity Questionnaire, and (C) Patient Health Questionnaire–9.

From June 2017 to January 2018, we consecutively assessed 1247 patients who underwent breast cancer surgery for study eligibility (Figure 1). After screening, 591 patients were excluded, 176 patients could not be contacted during the hospital stay, and 320 patients refused to join the study. A total of 160 patients were enrolled in this study. Among them, 54 patients did not complete the mobile app survey within 14 days after the baseline date. Thus, we included 106 patients who responded to the app survey at least once within the defined time period. The example of app screenshots of the survey is shown in Figure 2.

Table 1 summarizes the demographic and clinical characteristics of the subjects as absolute and relative frequencies. The subjects were aged 44.9 (SD 7.1) years. Among the patients, 77.4% (82/106) were aged less than 50 years, 64.2% (68/106) had an educational attainment of college level or higher, and 46.2% (49/106) were currently employed. Among breast cancer stages, 10.4% (11/106) of patients had stage 0, 45.3% (48/106) had stage I, 26.4% (28/106) had stage II, and 17.9% (19/106) had stage III disease (Table 1). Fifty patients completed adjuvant or neoadjuvant chemotherapy before beginning the data collection.

**Table 1:** Subject demographics (n=106).

Characteristic		Value
**Age in years, mean (SD)**		44.9 (7.1)
	<50, n (%)	82 (77.4)
	≥50, n (%)	24 (22.6)
**Marital status, n (%)**		
	Married	90 (84.9)
	Single	14 (13.2)
	Others	2 (1.9)
**Education, n (%)**		
	≤High school	38 (35.8)
	>High school	68 (64.2)
**Employed, n (%)**		
	Yes	49 (46.2)
	No	57 (53.8)
**Comorbidity, n (%)**		
	Yes	72 (67.9)
	No	34 (32.1)
**Past episode of depression, n (%)**		
	Yes	1 (0.9)
	No	101 (95.3)
	No response	4 (3.8)
**Surgery, n (%)**		
	Mastectomy	7 (6.6)
	Breast-conserving surgery	74 (69.8)
	Mastectomy with reconstruction	25 (23.6)
**Chemotherapy, n (%)**		
	Yes	50 (47.2)
	No	56 (52.8)
**Antihormonal therapy, n (%)**		
	Yes	86 (81.1)
	No	20 (18.9)
**Radiation therapy, n (%)**		
	Yes	86 (81.1)
	No	20 (18.9)
**Targeted therapy, n (%)**		
	Yes	98 (92.5)
	No	8 (7.5)
**Stage, n (%)**		
	0	11 (10.4)
	I	48 (45.3)
	II	28 (26.4)
	III	19 (17.9)
**Distress thermometer, n (%)**		
	Score of 5 or higher	35 (33.0)
	Score of less than 5	71 (67.0)
**PHQ-9^a^ total score, n (%)**		
	Score of 11 or higher	23 (21.7)
	Score of less than 11	83 (78.3)

^a^PHQ-9: Patient Health Questionnaire–9.

### Validating the Mobile App Survey

Among 106 patients, 102 patients completed the weekly app survey (DT and IPAQ) within 7 days after the paper survey visit. Among them, 73 patients completed the weekly app survey within 3 days after the paper survey visit. In addition, 34 patients responded to the app-based questionnaire on the same day that they answered the paper-based questionnaire (Table 2). Of the 106 patients, 96 responded to the app-based questionnaire within 14 days of completing the paper-based questionnaire (PHQ-9); 63 patients answered within 7 days, 39 within 3 days, and 18 patients answered the app-based questionnaire on the same day that they answered the paper-based questionnaire.

**Figure 3:**
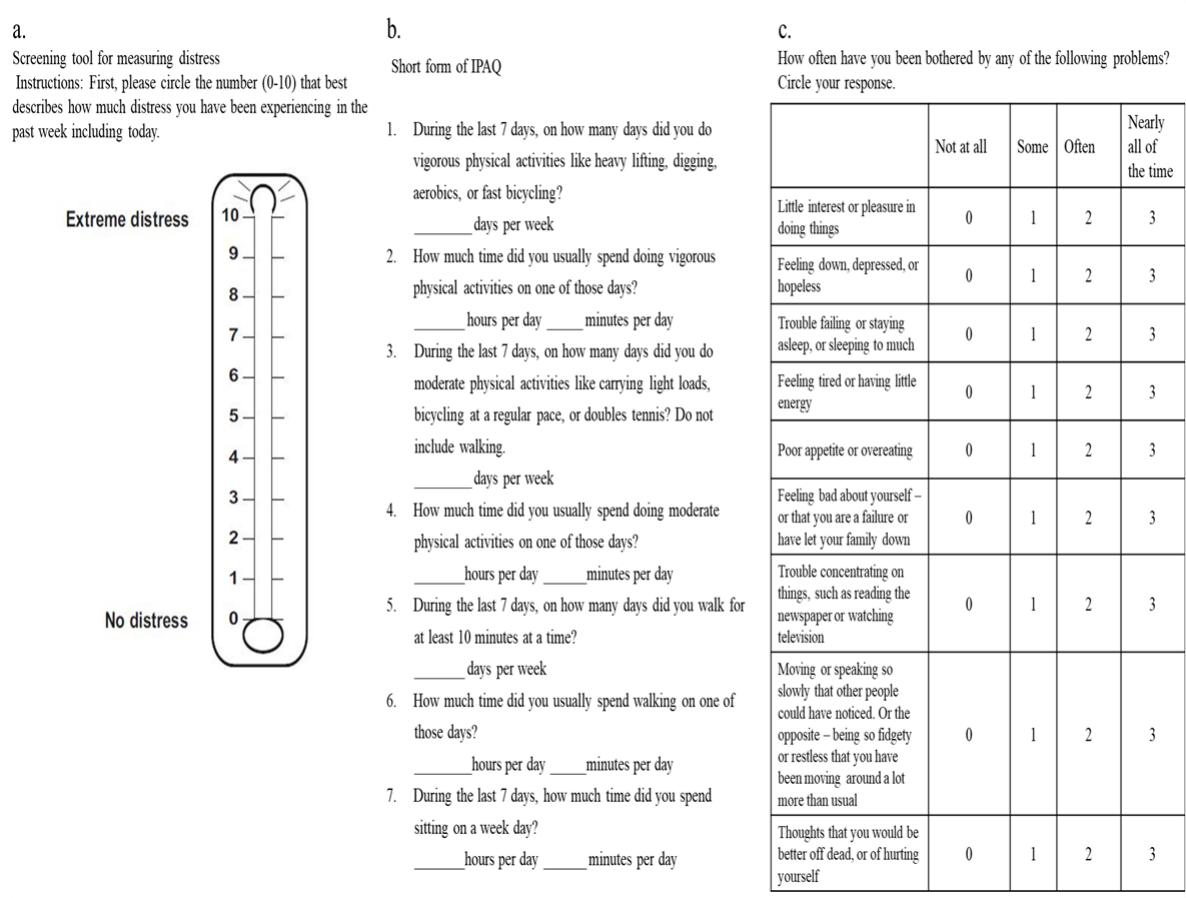
Paper-based versions of the (A) Distress Thermometer, (B) International Physical Activity Questionnaire, and (C) Patient Health Questionnaire–9.

The screening tool for measuring distress gives a numerical representation of the degree of distress (Figure 3A), IPAQ (Q2-6) asks about activity levels, and IPAQ (Q7) asks about time spent sitting (Figure 3B). IPAQ (Q2-6) and IPAQ (Q7) were analyzed separately in Table 2 because the contents of each question are different.

Spearman correlation values between the average values in app-based questionnaire being responded to within 7 days, within 3 days and on the same day and the values in the baseline paper survey were .415 (*P*<.001), .437 (*P*<.001), and .603 (*P*<.001), respectively, and the correlation values for IPAQ (Q2-6) were .291 (*P*=.003), .324 (*P*=.005), and .427 (*P*=.01), respectively. These were all statistically significant.

However, the correlation values of IPAQ (Q7) were .061 (*P*=.54) and .090 (*P*=.45) within 7 days and 3 days, respectively, and were not statistically significant. The correlation value, responded on the same day as the paper survey, was .155 (*P*=.38), which was also statistically insignificant (Table 2).

In terms of required sample size (Table 3), all required sample sizes to validate the correlation between the weekly app survey and corresponding paper survey were smaller than our sample sizes (102, 73, and 34) for DT. For IPAQ total physical activity metabolic equivalent of task (MET; Q2-6), the required sample sizes were slightly larger than our sample size. Even though our sample sizes were slightly smaller than the required sample sizes, the correlation coefficients were statistically significant.

**Table 2:** Spearman correlation coefficients between the value on the paper survey and the average value on the weekly app survey.

Survey	Days before and after the paper survey, number of patients reporting through the app, and scores										
	Same day (n=34)			3 days (n=73)				7 days (n=102)			
	Correlation	S-statistic	*P* value	Correlation	S-statistic	*P* value	Correlation		S-statistic	*P* value	
Distress thermometer	.603	2601	<.001	.437	36,511	<.001	.415		103,473	<.001	
IPAQ^a^ total physical activity MET^b^ (Q2-6)	.427	3753.1	.01	.324	43,790	.005	.291		125,385	.003	
IPAQ sitting MET (Q7)	.155	5530.3	.38	.090	58,977	.45	.061		166,062	.54	

^a^IPAQ: International Physical Activity Questionnaire.

^b^MET: metabolic equivalent of task.

**Table 3:** Sample size and actual statistical power in the correlation analysis of weekly app survey.

Survey	Days before and after the paper survey and scores							
	Same day			3 days			7 days	
	Sample size	Actual power	Sample size		Actual power	Sample size		Actual power
Distress Thermometer	21	.955	52		.952	58		.952
IPAQ^a^ total physical activity MET^b^ (Q2-6)	50	.962	98		.950	123		.951
IPAQ sitting MET (Q7)	446	.950	1331		.950	2904		.950

^a^IPAQ: International Physical Activity Questionnaire.

^b^MET: metabolic equivalent of task.

**Figure 4:**
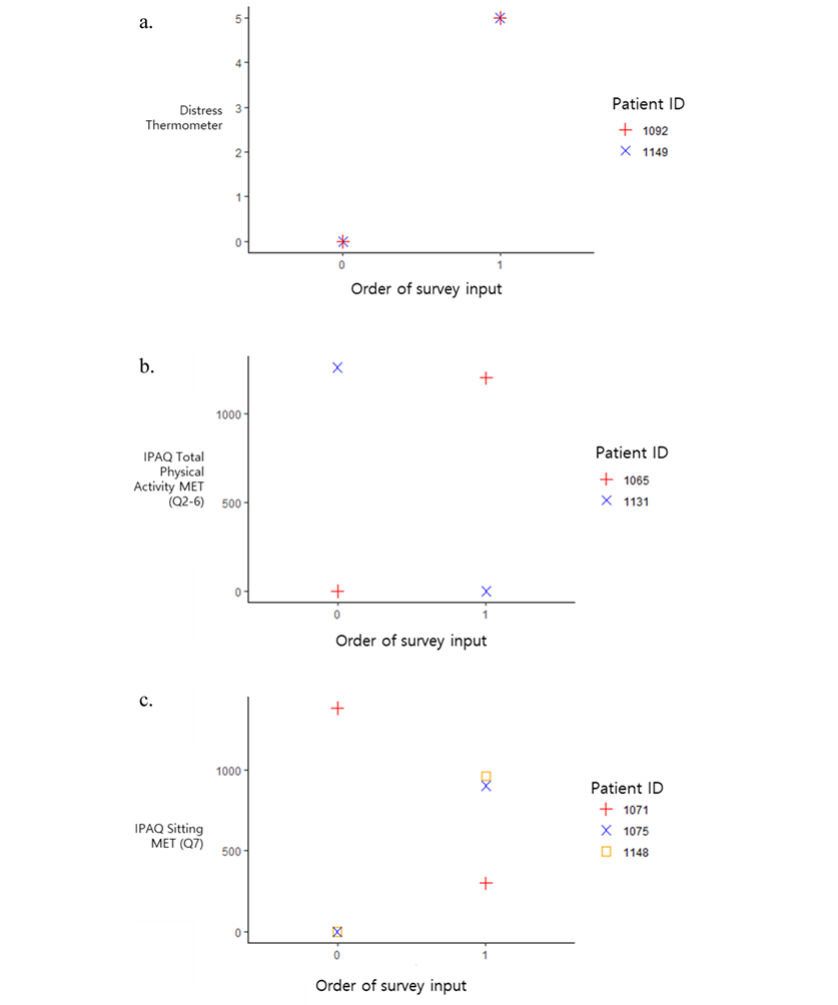
Examples of app data for (A) Distress Thermometer, (B) International Physical Activity Questionnaire Q2-6, and (C) International Physical Activity Questionnaire Q7.

For IPAQ sitting MET (Q7), the required sample sizes are relatively large since the correlation values are small. Thus, we can say that if we collect more data from a larger sample, the correlation between the paper survey and app survey of IPAQ Q7 can be significant. Even though the insignificance of correlation is due to the small sample size, the correlation values are also small (.061, .090, and .155), and thus we can say that the IPAQ Q7 shows different patterns in the paper-based survey and app survey. Figure 4 shows a graph of patient survey results with the largest difference in the values among those who surveyed more than once in 15 days, considering a week before and after the paper survey. There were increased or decreased trends in the app values, and there could be a gap between the values in the paper survey (single point) and the values in the app survey (the average of multiple points).

Concordance correlation analysis, represented by the value C_b, in Table 4 also shows similar patterns with the previous correlation analysis. For DT and IPAQ (Q2-6), the C_b is larger than .90, which means that the values from the app-based survey are at least moderately matched with the paper-based survey if the values of app survey are recorded within 3 days before and after the paper survey. By contrast, for IPAQ (Q7), the C_b is smaller than .90, which means a poor association with the paper-based survey regardless of the time range of app inputs. Thus, we found that the app-based survey can substitute for the paper-based survey for DT and IPAQ (Q2-6), while the association between app-based inputs and paper survey of sitting time (IPAQ Q7) shows a poor association.

In Table 5, we show the coefficients on the value in the app survey and the odds ratio using ordered logistic regression. Like the results in the previous correlation analysis, for DT and IPAQ total physical activity MET (Q2-6), the coefficients on the value in the app survey are all significant (*P*<.001), while the coefficients on app values for IPAQ sitting MET (Q7) are not significant for 3-day and 7-day intervals.

**Table 4:** Concordance correlation coefficients C_b between the value in the paper survey and the average value in the weekly app survey.

Survey	Days before and after the paper survey and number of patients reporting through the app		
	Same day (n=34)	3 days (n=73)	7 days (n=102)
Distress thermometer	.962	.901	.870
IPAQ^a^ total physical activity MET^b^ (Q2-6)	.988	.936	.776
IPAQ sitting MET (Q7)	.688	.823	.799

^a^IPAQ: International Physical Activity Questionnaire.

^b^MET: metabolic equivalent of task.

**Table 5:** Association between the value in the paper survey and the average value in the weekly app survey.

		Average value in the app survey				AIC^a^	Observations
		Beta	OR^b^	Z value	*P* value		
**Distress thermometer**							
	Same day	0.812	2.252	4.121	<.001	154.95	34
	3 days	0.472	1.603	4.230	<.001	366.73	73
	7 days	0.513	1.670	4.775	<.001	498.66	102
**IPAQ^c^ total physical activity MET^d^**							
	Same day	0.457	1.579	3.384	<.001	239.47	34
	3 days	0.324	1.383	4.753	<.001	533.03	73
	7 days	0.304	1.355	5.086	<.001	783.43	102
**IPAQ sitting MET**							
	Same day	0.003	1.003	2.631	.009	182.56	34
	3 days	0.001	1.001	1.296	.20	412.78	73
	7 days	0.001	1.001	1.363	.17	577.75	102

^a^AIC: Akaike information criterion.

^b^OR: odds ratio.

^c^IPAQ: International Physical Activity Questionnaire.

^d^MET: metabolic equivalent of task.

Table 6 shows the relationship between the answers of PHQ-9 (Q1-9) and PHQ (Q10) paper-based questionnaires and the answers of the corresponding app-based questionnaires. We analyzed the association between the paper-based questionnaires and the cases wherein the app-based questionnaires, answered on the same day when the paper-based questionnaire was answered or within 3, 7, or 14 days from the day. The PHQ-9 (Q1-9) questionnaires are standard format as shown in Figure 3C. In addition to PHQ-9 (Q1-9), PHQ (Q10) asks “if you checked off any problems among PHQ-9 (Q1-9), how difficult have these problems made it for you to do your work, take care of things at home, or get along with other people?”

According to the time interval between the paper-based questionnaire and the app-based questionnaire, the Spearman correlation values of the PHQ-9 sum value were .469 for 14 days, .574 for 7 days, .593 for 3 days, and .512 for the same day. Similarly, the correlation values for the answer to question 10 were .283 within 14 days, .409 within 7 days, .415 within 3 days, and .736 on the same day. Roughly speaking, the shorter the interval between the paper-based questionnaire and the app-based questionnaire, the higher the correlation value. All *P* values were statistically significant (Table 6).

**Table 6:** Spearman correlations between the values on the paper survey and the average values on the biweekly app survey.

Survey	Days before and after the paper survey, number of patients reporting through the app, and scores											
	Same day (n=18)			3 days (n=39)			7 days (n=63)			14 days (n=96)		
	Correlation	S-statistic	*P* value	Correlation	S-statistic	*P* value	Correlation	S-statistic	*P* value	Correlation	S-statistic	*P* value
PHQ-9^a^ sum	.512	472.93	.03	.593	3720.6	<.001	.574	17,767	<.001	.469	78,362	<.001
PHQ-10^b^	.736	256.09	.001	.415	5345.6	.009	.409	24,640	.001	.283	105,715	.005

^a^PHQ-9: Patient Health Questionnaire–9.

^b^PHQ-10: Patient Health Questionnaire Q10.

In Table 7, the required sample sizes are close to our sample size or slightly larger than our sample size. Even though some of our sample sizes were slightly smaller than the required sample sizes, the correlation coefficients were statistically significant. In addition, the actual powers are also sufficient as larger than .95.

In Table 8, we found that the app-based survey highly associated with the paper-based survey for the PHQ-9 sum value and PHQ (Q10), as the values of CCC are either close to .90 or larger than .90.

In Table 9, we show the coefficients on the value in the biweekly app survey and the odds ratio using ordered logistic regression. Like the results in the previous correlation analysis, for PHQ-9 sum and PHQ (Q10), the coefficients on the value in the app survey are all significant (*P*<.05). For PHQ-9 sum, the coefficients are smaller than 1, which means that the values in the app survey tend to be larger than the values in the paper survey. By contrast, for PHQ (Q10), the coefficients are larger than 1, which means that the values in the app survey tend to be smaller than the values in the paper survey.

**Table 7:** Sample size and actual statistical power in the correlation analysis of biweekly app survey.

Survey	Days before and after the paper survey and scores										
	Same day		3 days			7 days			14 days		
	Sample size	Actual power		Sample size	Actual power		Sample size	Actual power		Sample size	Actual power
								
PHQ-9^a^ sum	36	.951		26	.955		28	.954		44	.951
PHQ-10^b^	15	.960		58	.952		60	.952		130	.950

^a^PHQ-9: Patient Health Questionnaire–9.

^b^PHQ-10: Patient Health Questionnaire Q10.

**Table 8:** Concordance correlation coefficients C_b between the value in the paper survey and the average value in the biweekly app survey.

Survey	Days before and after the paper survey, number of patients reporting through the app, and scores			
	Same day (n=18)	3 days (n=39)	7 days (n=63)	14 days (n=96)
				
PHQ-9^a^ sum	.941	.967	.901	.894
PHQ-10^b^	.940	.938	.879	.936

^a^PHQ-9: Patient Health Questionnaire–9.

^b^PHQ-10: Patient Health Questionnaire Q10.

**Table 9:** Association between the value in the paper survey and the average value in the biweekly app survey.

Survey		Average value in the app survey				AIC^a^	Observations
		Beta	OR^b^	Z value	*P* value		
**PHQ-9^c^ sum**							
	Same day	0.437	1.548	2.504	.01	25.12	18
	3 days	0.296	1.344	3.655	<.001	192.35	39
	7 days	0.282	1.326	4.402	<.001	360.71	63
	14 days	0.247	1.280	5.253	<.001	544.75	96
**PHQ-10** ^d^							
	Same day	2.069	7.917	2.556	.01	20.02	18
	3 days	2.168	8.741	2.885	.004	56.65	39
	7 days	2.155	8.628	3.433	.001	103.13	63
	14 days	1.343	3.831	3.679	<.001	169.69	96

^a^AIC: Akaike information criterion.

^b^OR: odds ratio.

^c^PHQ-9: Patient Health Questionnaire–9.

^d^PHQ-10: Patient Health Questionnaire Q10.

## Discussion

### Principal Findings

As we are now able to diagnose and treat cancer early, the number of cancer survivors has increased worldwide. As the number of cancer survivors is increasing, we should pay more attention to them. Distress screening is a particularly important screening test for cancer survivors. According to the statistics of breast cancer patients, the prevalence of depression and anxiety among breast cancer survivors is 22% and 10%, respectively [16]. However, it is not easy for a clinician to diagnose a patient’s stress early. Most cancer specialists do not have enough time to see their patients as they lack resources such as manpower, finances, and time to screen for pain, anxiety, or depression. In recent psychiatric research, a digital footprint created passively through mobile technology has been used as a tool for remote monitoring of patients. The feasibility of using data collected from mobile devices for developing a new measure of mental health has been established in several studies [17].

The most important aspect in collecting clinical data is the speed and reliability of data collection. Mobile technologies, which are widely used around the world, are also widely used in medical field and health care because they add a significant positive aspect of cost-effectiveness in managing data and improving the quality of clinical results [18]. Mobile health is a “medical and public health practice supported by mobile devices, such as mobile phones, patient monitoring devices, personal digital assistants, and other wireless devices” as reported by the World Health Organization. If an electronic questionnaire is used instead of a paper-based questionnaire, the robustness of the electronic questionnaire must be established according to international guidelines first. This means that an equivalent measurement robustness should be demonstrated between both questionnaires [19].

The purpose of this study was to develop an electronic version of an original paper-based questionnaire and validate it. The electronic questionnaire retains the questions of the original paper-based questionnaire; however, the layout has been adapted for use on smartphones. The app was developed keeping in mind the ease of use not only for patients who have experience with smartphones but also for those who are new to smartphones. In the process of making a paper-based questionnaire into an electronic questionnaire, it is unclear which correction is being referred to. Because of this correction process, equivalence analysis between the electronic questionnaire and paper-based questionnaire needs to be conducted. To create the app used in this study, we needed to adjust the ePRO appropriately. We adjusted the size of the text and added a ScrollView feature that enables scrolling down to see all the sentences. This calibration process is why equivalence analysis is necessary. No significant difference was found between the two approaches for all the items investigated. This result corroborates previous studies stating that there were few or no differences between the electronic and paper-based questionnaires [20,21]. In a previous study, Bierbrier et al [22] did not focus on measuring instruments related to the musculoskeletal system but experimented with various mHealth apps from the Google Play Store and App Store to assess the accuracy of the electronic physiotherapy questionnaire using a smartphone.

It is important that the data obtained from the app is accurate. Therefore, the possibility of errors due to human involvement should be eliminated. There is some evidence to support the promising role of a mobile app to remotely collect PRO, as the mobile app-based PRO questionnaires could be free from generating a bias driven by Hawthorne effect. Bush et al [23] evaluated active military personnel in the United States and administered a 7-question questionnaire via an app and on paper, and the responses were similar. Garcia-Palacios et al [24] have also conducted a study on the use of questionnaires in patients with fibromyalgia. In this study, the mean pain and fatigue scores reported in paper and smartphone questionnaires were not statistically significantly different. ePRO for patients has been found to be in good agreement with the results of the paper version [18]. In a study on the validity and reliability of International Prostate Symptom Score, Kim et al [25] reported a strong correlation between results of smartphone and paper-based questionnaires.

It is important to be able to receive useful information data through ePRO. It is possible to collect data several times a day whenever the patient wants. In this way, we can collect continuous data over time and use this database to create a more strategic and systematic treatment plan for doctors to treat cancer survivors. In addition, it not only benefits patients but also significantly changes and advances the overall health and health care system [18].

When using questionnaires to assess health outcomes by electronic methods can provide more accurate and efficient information than paper-based methods, and since the information is communicated through mobile phones, the answers can be safely achieved. This suggests that patients may be better served electronically than with paper [18]. It has also been reported that responding to a questionnaire using a mobile phone is faster and safer than it is using a paper-based questionnaire [25,26].

These results suggest that mobile devices have a significant potential as tools for distress screening in patients with unmet needs. In agreement with previous reports, during the 6-month study of 106 patients who participated in this study, breast cancer patients collaborated very well with data collection using smartphone apps and maintained a high level of compliance. Data for the DT questionnaire, which is answered every week, and the PHQ-9, which is answered every 2 weeks, were collected through the same app, and overall collection rates were 42.42% (3597/8480) and 41.86% (1775/4240), respectively. This suggests that ePRO has a greater effect on improving patient lifestyle than paper or web-based methods [27].

In our study, the Spearman correlation between the paper-based questionnaire and app-based questionnaire is distributed between .30 and .80 depending on the answer interval, except the IPAQ (Q7; sitting MET). The shorter the time interval was, the larger the value.

Even in the same patient, since emotional factors such as distress change frequently, DT (Figure 4A) also changed depending on the answer time. Overall, although the paper-based questionnaire is admittedly a standard test, a smartphone app-based questionnaire can be a better way to identify patient conditions that change from time to time and incorporate the findings into a treatment plan.

### Limitations

Our research has some limitations. First, we did not evaluate the experience and familiarity with smartphone use of patients in the study. A previous study has reported that patients found completing the ePRO questionnaire more comfortable and better than completing the paper-based questionnaire [19]. We also did not consider the time it takes to complete the survey. A previous study has reported that answering a paper-based questionnaire takes less time than answering an app-based questionnaire [19].

In addition, all subjects in this study were Android users. Thus, one may have a concern on the sample selection bias driven by the focus on an Android app. However, iPhone penetration in the elderly in Korea is not very great, thus the selection bias is not serious in this study. Finally, the subjects in this study are younger than the total breast cancer patient population as we recruited a sample that could use a smartphone. Thus, the effectiveness of a mobile app to collect patient-reported information for older patients should be validated in a future study.

Despite these limitations, our study is the first empirical effort to demonstrate the effectiveness of a smartphone app-based version of a questionnaire associated with distress and physical activity using a proper statistical analysis. Using electronic methods to measure the PROs of distress and physical activity can help doctors and patients to more effectively collect the information. Doctors can evaluate distress and physical activity, improve their clinical observation, and make better decisions related to treatment at the time of patient consultation. In addition, there is a growing interest in patient-centered care, which shows that patients participate in their health care and improve their health. Therefore, having the opportunity to manage distress and physical activity at home, and evaluate the results with doctors has the advantage of strengthening the relationship between patients and doctors.

### Conclusions

The app-based questionnaire related to stress and physical activity is a useful assessment tool for health care professionals. Using the app, clinicians can easily collect answers to questionnaires from their patients and effectively manage, store, and organize them. The app shows a high level of validity and compliance for DT, IPAQ (Q2-6), and PHQ-9. In conclusion, the app version of the questionnaire was not inferior to the paper version, and there was sufficient potential for equal use in clinical practice.

## Appendix

Multimedia Appendix 1 Korean language version of the (A) Distress Thermometer, (B) International Physical Activity Questionnaire, and (C) Patient Health Questionnaire–9.

## Abbreviations

CCC: concordance correlation coefficient
DT: Distress Thermometer
ePRO: electronic patient-reported outcome
IPAQ: International Physical Activity Questionnaire
MET: metabolic equivalent of task
mHealth: mobile health
PHQ-9: Patient Health Questionnaire–9
PRO: patient-reported outcome
