# Supplementary figures and images for: A Mobile Technology for Collecting Patient-Reported Physical Activity and Distress Outcomes: Cross-Sectional Cohort Study
Source: JMIR Mhealth Uhealth. 2020 May 4;8(5):e17320. doi: 10.2196/17320 (PMC7235805; doi:10.2196/17320)

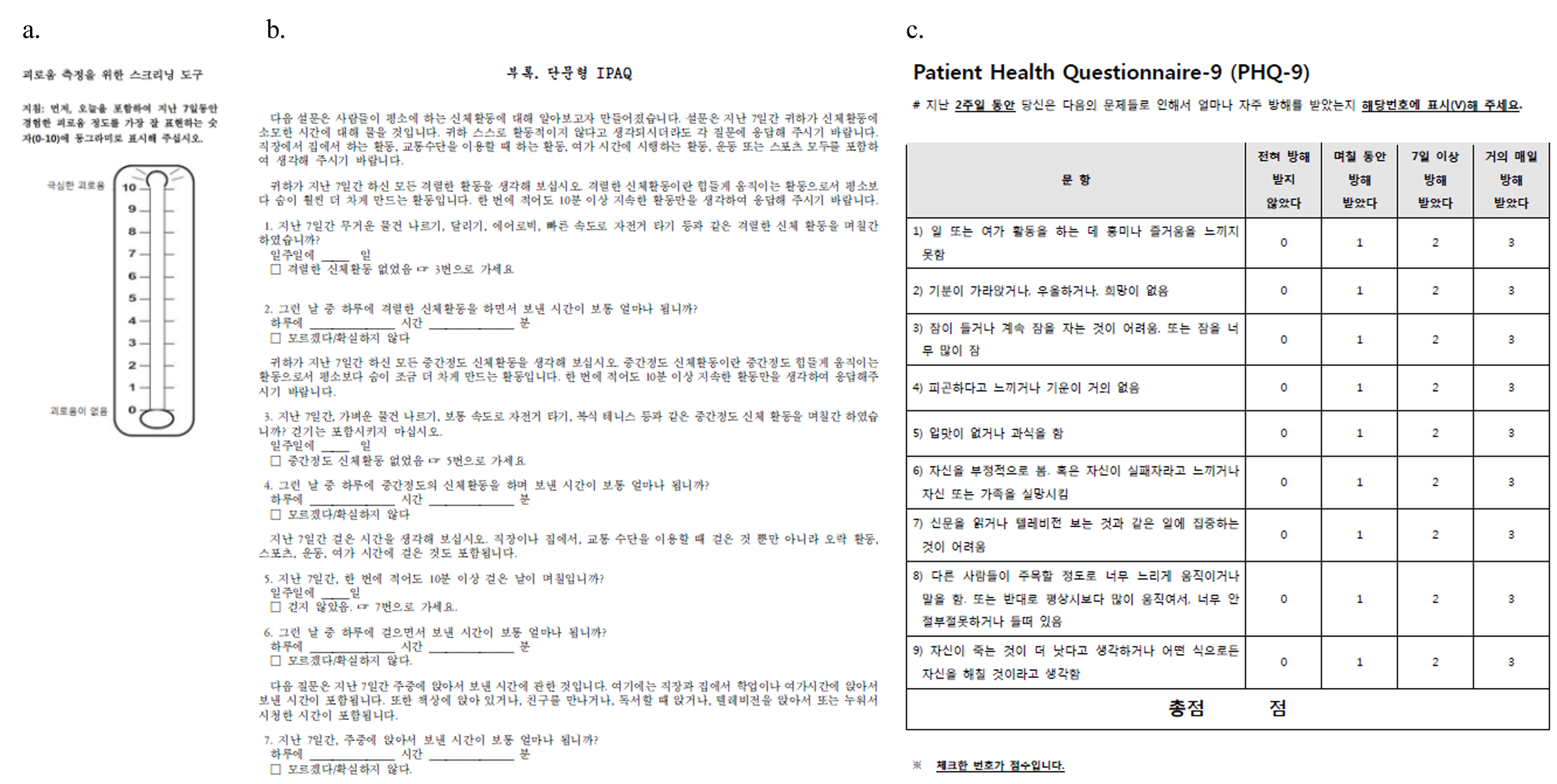

Supplement: Multimedia Appendix 1 [file mhealth_v8i5e17320_app1.png]
